# VA physicians intent to leave and correlations to drivers of burnout: a cross-sectional study

**DOI:** 10.1186/s12913-024-12079-5

**Published:** 2025-01-23

**Authors:** Robert C. Oh, David C. Mohr, Tamara M. Schult

**Affiliations:** 1 Department of Veterans Affairs, Palo Alto Health Care System, 3801 Miranda Ave., Palo Alto, CA 94304 USA; 2 Department of Medicine and Division of Primary Care and Population Health, Stanford University School of Medicine, 300 Pasteur Drive, S-102, Stanford, CA 94305 USA; 3 Veterans Health Administration National Center for Organization Development, 4605 Duke Drive, Suite 800, Mason, OH 45040 USA; 4 Department of Health Law, Policy & Management, Boston University School of Public Health, Boston, MA USA; 5 Department of Veterans Affairs Office of Patient Centered Care & Cultural Transformation, 810 Vermont Avenue NW, Washington D.C., 20420 USA

**Keywords:** Burnout, Professional / etiology, Burnout, Professional / epidemiology, Burnout, Psychological, Health workforce, Physicians, Intention, Personnel turnover

## Abstract

**Background:**

Physician well-being and workforce retention within the healthcare system is of critical importance. Understanding physicians’ intent to leave the organization will inform efforts on optimizing the physician workforce. In this study, we examine the association of burnout and specific drivers of burnout on turnover intentions.

**Methods:**

The study was a cross-sectional design using data collected from an organization-wide workforce survey. The sample included 16,363 respondents from the Veterans Health Administration (VA). A multinomial model was run to compare physicians indicating turnover intent because they were: a.) changing internal jobs; b.) leaving the organization for another job; or c.) retiring, against physicians indicating they had no plans to turnover. Explanatory variables in the model included burnout, drivers of burnout, and demographics. We also asked about the primary reason behind turnover intent.

**Results:**

Most physicians responding to the survey (n = 13,083, 80%) indicated they would stay within their current job, while 5.8% indicated they would look for another VA job, 5% indicated planning to retire, and 9.3% said they would leave the VA workforce altogether. Burnout and less favorable senior leadership perceptions were associated with greater odds of turnover intent specific to finding another VA job, leaving VA, or retire. Experiencing discrimination was related to turnover intent for another VA job and leaving VA, while satisfaction with workload and recognition were related to lower odds of finding another VA job or leaving VA. Culture of well-being was associated with lower odds of leaving VA.

**Conclusions:**

The study highlights how burnout is associated with turnover intentions with physicians have differing rationales for leaving. Several drivers of burnout were related to turnover intentions for both finding another VA job and leaving VA altogether. Efforts to improve workforce well-being and drivers of burnout may help address the different rationales physicians may have for considering leaving.

**Supplementary Information:**

The online version contains supplementary material available at 10.1186/s12913-024-12079-5.

## Background

The Veterans Health Administration (VA) is the largest healthcare delivery system in the United States (U.S.) – responsible for the healthcare needs of over 9 million enrolled Veterans. Physician burnout within VA is of critical importance. Work-related burnout can lead to negative organizational outcomes, including higher employee disengagement, turnover, absenteeism, and lower productivity, which can adversely affect access and quality of care [1–3]. Burnout in the clinical workforce can also lead to early retirement and change of jobs [4, 5]. Like many other healthcare systems both during and since the COVID-19 pandemic, VA saw an increase in workplace burnout and its associated impacts including increased turnover. Employees attritted from the VA at a rate of 11% in fiscal year 2022 which was higher than the previous average of 9.8% [6]. Burnout has been correlated to workplace departures and intent to leave an institution. Medscape’s annual physician survey on burnout found that 49% of physicians surveyed reported burnout [7]. Up to 25% of physicians reported changing work settings or getting a different job to tackle burnout. Physician departure in medicine exacerbates the recognized physician shortage as our population grows and ages [4].

There is limited research on physicians’ intent to leave their institution. A recent study demonstrated that academic physicians’ intent to leave the institution was correlated to supervisor behaviors, peer support and organizational values alignment [8]. However, using intent to leave data may not always provide information on why respondents want to leave. For example, some individuals may be considering turnover for retirement, or to get a promotion within another part of the company. Thus, understanding not only whether respondents are considering leaving but where they plan to go and factors behind that decision would be an important extension for research understanding. In the VA, there have been no studies looking explicitly at why physicians leave the organization. This critical gap in the literature and understanding why physicians leave the largest healthcare organization could inform further strategy on retention and recruitment of physicians for the U.S. healthcare system more broadly.

Maslach and Leiter proposed six areas of work-life that correlate with either professional fulfillment or burnout including workload, control, reward, community, fairness and values [9]. While burnout focuses on the negative impacts of work, professional fulfillment focuses on positive aspects of the role and work. Professional fulfillment refers to the degree of intrinsic positive reward derived from work, including happiness, meaningfulness, contribution, self-worth, satisfaction, and feeling in control when dealing with difficult problems at work [10]. Each area of work-life, if optimized, can lead to professional fulfillment, but if not optimized, can lead to burnout.

In this study, we hypothesize that VA physicians’ intent to leave will be associated with specific drivers of burnout reflecting the areas of work life. A better understanding of the particular drivers of burnout that relate to physician intent to leave will allow the organization to design and implement more tailored, comprehensive interventions addressing both system and individual-level factors impacting ability to retain physicians and mitigate burnout. We also extend prior work by examining the different motivations behind turnover intentions and how they may be influenced by drivers of burnout.

## Methods

### Study

The study was conducted as a cross-sectional observational design. Data was obtained from an administrative database with survey responses from employees within VA.

### Ethics approval and consent to participate

The scope of work for the study was reviewed by the VA Palo Alto Institutional Review Board and Stanford University as Human Subjects Research and determined to meet the exemption criteria for non-research activities and received a waiver for ethical review (Protocol 73818). Informed consent was waived by the committees as the analysis used a secondary dataset already collected.

### Sample

The study sample was obtained using responses to the All Employee Survey (AES) conducted in June 2023. The AES is routinely administered to all respondents across the Department of Veterans Affairs for the purpose of organizational improvement [11, 12]. Respondents are mapped to workgroups by a facility coordinator and the survey is promoted by facility leadership. Regular reminders for survey completion and progress are distributed by email. Multiple methods are available for responding to the survey; however, 99% of respondents answered using an online survey link. An independent vendor administered the survey. An internal VA team conducted data cleaning. The survey consists of approximately 20 demographic items, 60 core items, plus additional module-based questions added by programs within VA.

A total of 293,164 VHA employees responded to the survey (74.3%). The study sample included employees who indicated their occupation was one of several possible physician roles. Because we were focused on understanding why physicians may be considering leaving, we excluded respondents who indicated leaving for unspecified causes or had missing values leaving us with 16,363 respondents for the analysis at 140 VA Medical Centers, 18 regional network offices, and 18 national program offices.

### Measures

Using the AES, we created both the dependent variable (pertaining to intention to leave) and independent variables (demographics, site characteristics, burnout, and burnout drivers). The dependent variable of intent to leave was asked as “Are you considering leaving your job within the next year, and if so why?” Responses were characterized into four categories: No; taking another job within VA; leaving VA (for a job either within or outside of the Federal government); and retiring. Among respondents who indicated they were considering leaving, they were also asked “What is the primary factor that has led you to consider leaving your current position?” with a choice of nine different response options. Items and response options used in the study appear in Supplemental Appendix A.

### Independent variables

#### Burnout

*Burnout* was coded based on the Maslach Burnout Inventory [13] and consisted of two commonly used and validated items on emotional exhaustion and depersonalization [14, 15]. Respondents indicated how often they felt that way on a seven-point scale ranging from “never” to “every day.” Responses of “once a week” or more for either item were coded to reflect burnout*.*

#### Drivers of burnout

We used a single item from the survey that assessed the reasonable workload driver. The employee control driver was represented using a support for work-family balance item. The rewards and recognition driver was based on two items asking about recognizing performance differences and job recognition (α = 0.81). The community driver was represented with an item assessing discrimination, three items on workplace civility (α = 0.90), and four items on culture of well-being (α = 0.95) [10]. The fairness driver was based on a five-item supervisor behavior measure (α = 0.94). The senior leadership driver was based on five items (α = 0.95). Respondents rated most items noted on a five-point Likert scale ranging from “strongly disagree” to “strongly agree” or on a satisfaction scale in a few cases. Except for discrimination, measures were treated as continuous variables.

#### Demographics

Gender was coded as male, female, or other. Age was coded as less than 40 years of age, 40–49, 50–59, and 60 plus years of age. Ethnicity was coded as Hispanic or non-Hispanic. Race was coded using the standard five categories and a combined multi-racial category. Physicians indicated their specialty occupation at the start of the survey, which was coded as anesthesiology, medicine, primary care, surgery, psychiatrist, or other clinical specialty. Respondents indicated if they had a faculty appointment. Supervisory role was coded as none or team leader; first line supervisor; and manager or executive.

#### Site characteristics

A dichotomous variable for whether the respondent was located at a regional network or national program office rather than a medical center was also created. Using values from the Area Health Resources Files [16], we computed a rate for the number of physicians divided by population estimates using the 2021 County-level values. Values were assigned based on the county code of the VA Medical Center.

### Statistical analysis

We first reviewed descriptive characteristics of the sample and item responses from the AES. Missing values on demographics were assigned to an “unknown” value for conducting multivariate models. Omitted responses to AES drivers of burnout items were left missing. We conducted a multinomial model with three possible options for leaving compared against physicians who indicated no intent to leave. A random effects model was conducted to account for respondents nested within medical center as an intraclass correlation coefficient, which indicated 2% of variability in the intent to leave outcome was explained by variability between sites. Analyses were conducted using SAS software 9.4 (SAS Institute Inc., Cary NC).

## Results

Respondent characteristics (Table 1) indicated the majority was male (n = 8365, 58.3%), White race (67%), non-Hispanic (92.1%), held a faculty appointment (58.4%), and a non-supervisory role (71.7%). The most common age groups were between 40–49 (28.9%) and 50–59 (28.3%). There was variability in specialty representation: medicine specialties (29.9%), primary care (21%), surgical (12.9%), and psychiatrists (11.8%).

**Table 1:** Demographic characteristics of physician respondents from VHA (N = 16,363)

Characteristic	N (%)
Specialty	
Primary care	3438 (21)
Anesthesiologist	726 (4.4)
Medicine or specialty	4897 (29.9)
Surgery or specialty	1984 (12.1)
Psychiatrist	1929 (11.8)
Other clinical specialty	3389 (20.7)
Gender	
Male	8365 (58.3)
Female	5917 (41.2)
Other	70 (.5)
Age	
Under 40	2886 (18.2)
40–49	4589 (28.9)
50–59	4494 (28.3)
60 or greater	3925 (24.7)
Race	
American Indian or Alaskan Native	50 (.4)
Asian	3220 (24.4)
Black or African American	738 (5.6)
Multi-racial	291 (2.2)
Native Hawaiian or other Pacific Islander	49 (.4)
White	8365 (58.3)
Ethnicity	
Hispanic	1243 (7.9)
Non-Hispanic	14,436 (92.1)
Faculty appointment	
Yes	9464 (58.4)
No	6738 (41.6)
Supervisory role	
None	11,583 (71.7)
First line supervisor	2392 (14.8)
Manager or executive	2170 (13.4)
Location: National Program or Regional Network Office	620 (3.8)

Regarding intention to leave, a majority (n = 13,083, 80%) indicated they would stay within their current job, while 5.8% indicated they would look for another VA job, 5% indicated planning to retire, and 9.3% said they would leave the VA workforce (Table 2). Approximately 34% of physicians indicated they were experiencing burnout. Among respondents, 6.7% experienced discrimination of some type. AES measures of workplace civility, work/family balance and senior leadership engagement/values were generally rated highly favorably among respondents.

**Table 2:** Descriptive statistics of survey items

**Variable**	**N (%)**
Intention to turnover	
Stay within VA at current job	13,083 (80%)
Another VA job	944 (5.8%)
Leave government	1516 (9.3%)
Retire	820 (5%)
Burnout	5605 (34.4%)
Experienced discrimination	1060 (6.7%)
**Variable**	**M (SD)**
Culture of well-being	3.77 (1.02)
Workplace civility	4.32 (.80)
Reasonable workload	3.80 (1.20)
Work and family balance	4.29 (1.00)
Recognition	3.80 (1.05)
Supervisor satisfaction	4.29 (.90)
Senior leadership	3.98 (1.01)
Physicians per 100,000 population (County-level)	513.08 (326.59)

Among physicians who expressed an intention to leave, the most common reasons provided in the follow-up question were job-related (23.8%), personal (18.4%), and compensation/benefits (16%) (Table 3). Job-related reasons were the most common reason for those looking for another VA job, physicians leaving VA selected compensation (26.9%) most often, and among those who plan to retire, personal reasons (52.5%) was the most selected response.

**Table 3:** Primary reasons provided by intention to leave among physicians^a^

	Overall (n = 3252)	Another VA job (n = 940)	Leave VA (n = 1508)	Retire (n = 804)
Job-Related	775 (23.8)	218 (23.2)	381 (25.3)	176 (21.9)
Personal	598 (18.4)	114 (12.1)	62 (4.1)	422 (52.5)
Compensation and/or benefits	520 (16)	82 (8.7)	406 (26.9)	32 (4)
Leadership	409 (12.6)	128 (13.6)	234 (15.5)	47 (5.9)
Work/Life Flexibilities	376 (11.6)	159 (16.9)	143 (9.5)	74 (9.2)
Professional	292 (9)	125 (13.3)	156 (10.3)	11 (1.4)
Supervisor	176 (5.4)	74 (7.9)	73 (4.8)	29 (3.6)
Discrimination	67 (2.1)	23 (2.5)	36 (2.4)	8 (1)
Workgroup	39 (1.2)	17 (1.8)	17 (1.1)	5 (.6)

^a^Full-text of response options to the question: What is the primary factor that has led you to consider leaving your current position?” 1 = Compensation and/or benefits (e.g., salary, benefits), 2 = Work/Life Flexibilities (e.g., Teleworking, Alternative Work Schedule, other work/life accommodations), 3 = Job-Related (e.g., type of work, workload, burnout, boredom), 4 = Personal (e.g., focus on new interests, attend school, family needs, health), 5 = Professional (e.g., better career prospects, career change), 6 = Workgroup (e.g., clash with coworkers), 7 = Supervisor (e.g., clash with supervisors), 8 = Leadership (e.g., unhappy with senior leadership, unable to adjust to new management style or organizational direction), 9 = Discrimination (e.g., not being treated like others)

In multinomial models (Table 4, and Appendix B) which compared each intent to leave category against respondents who planned to stay at current job, burnout was associated with greater intent to leave among all three categories of intent to leave: change VA jobs (OR = 2.15, 95% CI = 1.77 – 2.61), leave VA (OR = 2.97, 95% CI = 2.50 – 3.52), and retire (OR = 2.45, 95% CI = 1.97 – 3.05).

**Table 4:** Multinomial regression model for turnover intentions and drivers of burnout among VA physicians in 2023 (n = 11,508)

	**Another VA job**		**Leave VA**		**Retire**		
	**OR**	**95% CI**	**OR**	**95% CI**		**OR**	**95% CI**
Burnout	2.15^*^	1.77 – 2.61	2.97^*^	2.50 – 3.52		2.45	1.97 – 3.05
Culture of well-being	.94	.83 – 1.06	.83*	.75—.93		.99	.86 – 1.15
Workplace civility	.98	.84 – 1.13	1.07	.94 – 1.22		.88	.72 – 1.08
Discrimination	2.11^*^	1.60 – 2.78	1.90^*^	1.49 – 2.44		.96	.63 – 1.45
Reasonable workload	.85^*^	.78—.93	.85^*^	.79—.91		1.03	.93 – 1.14
Work and family balance	1.03	.90 – 1.17	1.03	.92 – 1.15		.98	.83 – 1.16
Recognition	.81^*^	.71—.93	.70^*^	.63—.79		.97	.82 – 1.15
Supervisor satisfaction	.99	.98 – 1.00	1.00	.99 – 1.00		1.00	.99 – 1.01
Senior leadership	.75^*^	.66—.85	.64^*^	.58—.72		.80^*^	.68—.94

Note: Model reports fully adjusted values accounting for respondent demographics and site characteristics. ^*^indicates *p* < .05

Among drivers of burnout, experiences of discrimination were associated with greater likelihood to change jobs within VA (OR = 2.11, 95% CI = 1.60 – 2.78) and leave the VA for another job (OR = 1.90, 95% CI = 1.49 – 2.44). A stronger culture of well-being had a lower likelihood of physicians wanting to leave the VA for another job (OR = 0.83, 95% CI = 0.75—0.93). Physicians who were more satisfied with their workload were less likely to want to change jobs within VA (OR = 0.85, 95% CI = 0.78—0.93) or leave VA for another job (OR = 0.85, 95% CI = 0.79—0.91). When respondents reported greater recognition efforts, there was a lower likelihood of wanting to change jobs within VA (OR = 0.81, 95% CI = 0.71—0.93) or leave VA for another job (OR = 0.70, 95% CI = 0.63—0.79). Physicians with greater satisfaction with senior leadership reported lower likelihood to change jobs within VA (OR = 0.75, 95% CI = 0.66—0.85), leave the VA (OR = 0.64, 95% CI = 0.58—0.72) or retire (OR = 0.80, 95% CI = 0.68—0.94).

Age was a very strong predictor of retirement with the 50 – 59 group (OR = 12.26, 95% CI = 3.87 – 38.94) and 60 plus group (OR = 136.98, 95% CI = 44.09 – 425.59). Compared to primary care providers, anesthesiologists (OR = 1.63, 95% CI = 1.13 – 2.36) were also more likely to consider leaving VA jobs. Physicians with faculty appointments were more likely to consider leaving VA (OR = 1.18, 95% CI = 1.00 – 1.39). Compared to non-supervisors, managers and executives (OR = 2.30, 95% CI = 1.88 – 2.90) were more likely to consider finding another VA job or leaving VA (OR = 1.41, 95% CI = 1.12 – 1.78).

## Discussion

Overall, in this analysis, we found that 80% of physicians reported an intent to stay at their current job. Of the 20% with intent to leave in the next year, nearly half reported intending to leave the VA workforce altogether. The most common reason (27%) reported for leaving the VA workforce was related to compensation and/or benefits. Compensation of physicians can also be a factor for retaining physicians [4]. Notably in our study, we found that anesthesiologists were more likely to leave the VA workforce compared to primary care physicians. A recent study of faculty physicians’ intent to leave noted that anesthesiologists had the highest intent to leave as well as high levels of burnout [8]. In the VA, however, anesthesiologist intent to leave may be related to the relative lower compensation for anesthesiology as a specialty in the VA compared to the civilian sector [17]. Secondly, approximately 25% of physicians reported that “job related” factors accounted for their intention to leave the VA. In the survey, examples of job-related reasons included “type of work, workload, burnout, or boredom.” Administrative burdens, excessive workload and the electronic health record (EHR) systems are common drivers of burnout in the physician workforce and may contribute to the intention to leave the VA [7, 18]. Allowing optimal workload and improving work and EHR efficiency that allows physicians to practice at the top of their licenses may help reduce the job related stressors that often drive physician burnout [18]. Burnout was found in 34% of the physician workforce, and not surprisingly, those that reported burnout predictably reported higher intent to leave the VA, change VA jobs, or retire. Interestingly, those who had a faculty appointment were more likely to leave the VA. This may be related to types of work in either research or educational positions that may be unavailable at the VA. Opportunities for physician retention in VA suggest changes in pay scales, optimizing workload, decreasing administrative burdens and furthering clinical, educational and research opportunities with affiliated universities may impact future physician intent to leave.

We also found that physicians were more likely to change to another VA job if they were in a manager or executive position. Physicians in leadership roles may be interested in upward mobility and career paths in leadership or it may reflect a decision to move away from leadership. Either way, these findings suggest that physicians may benefit from coaching and mentoring for those who want to be selected for leadership positions or for those having difficulty with supervisory roles. On the contrary, physicians who are satisfied with senior leadership were less likely to consider leaving their position. This is a similar finding from a large sample of academic physicians [19].

As it pertains to the specific burnout drivers and related areas of work-life – physicians who provided higher ratings on senior leadership, a reasonable workload, and recognition for their work, were less likely to leave their VA job. This is consistent with research that these work-life areas may influence retention within an organization [8, 9, 18–20]. In contrast, those who experienced higher levels of discrimination were more likely to pursue another job within VA or leave the VA altogether. This continues to highlight the importance of workplace diversity, equity, and inclusion efforts.

Finally, workplace culture and an organizational culture of well-being has been described as a foundational program to support employees [21, 22]. Unique to this survey was inclusion of a module with items on a culture of well-being – which queried respondents about workplace attitudes and a culture supportive of self-care opportunities. We found that greater endorsement of a culture of well-being was related to higher intent to stay and lower intent to leave; thus, the measure may be a predictor for further exploration for physician retention.

In the fall of 2021, to help combat attrition and to improve retention, the VA launched the Reduce Burnout and Optimize Organizational Thriving (REBOOT) taskforce. The REBOOT taskforce addresses the six areas of work-life – workload, job control, recognition, workplace harmony, organizational values and fairness. REBOOT also identified seven priority focus areas – including maximizing job control, implementing a Chief Well-being Officer role, optimizing meeting practices, optimizing training and education, strengthening mental health support for employees, addressing inefficiencies and strengthening the culture of servant leadership [23]. The results of this study can inform the REBOOT taskforce, specifically looking at physician retention and intent to leave. Improving supervisor development, providing recognition, reducing inefficiencies of practice, and exploring areas of opportunities for clinical or academic practices are in line with REBOOT efforts and may help the VA retain their physician workforce. The results of this study can inform the REBOOT taskforce, specifically looking at physician retention and intent to leave. Improving supervisor development, providing recognition, reducing inefficiencies of practice, and exploring areas of opportunities for clinical or academic practices are in line with REBOOT efforts and may help the VA retain their physician workforce.

Strengths of the study included a greater than 70% response rate across VA which strengthens the confidence in generalizability to all VA physicians. However, this is a cross-sectional analysis, and causal relationships can’t be inferred. Also, the self-reported nature of the study precludes determining the true outcomes of those who reported an intent to leave their current position. Indeed, we found that actual attrition rates in VA were lower than intent to leave as reported on the AES. When compared to internal administrative data for October 2022 – September 2023, the total VA loss rate for physicians was 8.3% with 4.6% resignations, and 2.5% retirement, indicating that intent to leave rates as reported on the AES for physicians were higher than actual attrition rates. While this study may not be generalizable to the civilian physician workforce, this study suggests that physicians’ intent to leave may be related to the areas of work-life, which may apply to other large healthcare organizations.

Limitations of the study include using one year of survey data. Data were anonymous, so we were unable to obtain individual data to follow respondents over time and know if they left or stayed. We had limited information on the reason to leave given the discrete responses. We also acknowledge that the model we used for the drivers of burnout were limited to the questions available from the AES survey and is not validated. The caveats with using employee survey data apply. For example, the study data is subject to non-response bias, although with a relatively high response rate this limits this concern. Further, data may be subject to common methods variance; thus, additional linkable data sources would be helpful.

## Conclusion

In conclusion, in this cross-sectional survey, we found that the areas of work-life were associated with physicians’ intent to leave. Improving VA’s culture of well-being and increasing opportunities for recognition may be protective for physician retention and warrants further study. Improving efficiency of practice, optimizing workload and expanding opportunities with affiliated academic institutions as well as compensation should be explored further to mitigate potential turnover. Finally, understanding the role of the physician as a leader and supervisor, including needed support in that role, may help improve both role satisfaction and aid understanding of leadership behaviors that influence job satisfaction and intent to stay.

## Supplementary Information

Supplementary Material 1

Supplementary Material 2

## Data Availability

The datasets generated and/or analyzed during the current study are not publicly available due to ethical reasons but are available from the corresponding author on reasonable request. Portions of the data are publicly available on data.gov under the All Employee Survey (AES) 2022–2023 webpage.
