# Supplementary material for: VA physicians intent to leave and correlations to drivers of burnout: a cross-sectional study
Source: BMC Health Serv Res. 2025 Jan 23;25:125. doi: 10.1186/s12913-024-12079-5 (PMC11755878; doi:10.1186/s12913-024-12079-5)
Supplement: Supplementary file 1 — Supplementary Material 1 [file 12913_2024_12079_MOESM1_ESM.docx]

**Supplemental Appendix A**

**Survey items and response options from the AES**

Turnover intention.

Are you considering leaving your job within the next year, and if so why?

Response options: No; Yes but taking another job within VA; Yes to retire; Yes to take another job within the Federal government; Yes to take another job outside the Federal government; Yes other

Turnover Reason

What is the primary factor that has led you to consider leaving your current position?

Response options:

- Compensation and/or benefits (e.g. salary, benefits)
- Work/Life Flexibilities (e.g. Teleworking, Alternative Work Schedule, other work/life accommodations
- Job-Related (e.g. type of work, workload, burnout, boredom)
- Personal (e.g. focus on new interests, attend school, family needs, health)
- Professional (e.g. better career prospects, career change)
- Workgroup (e.g. clash with coworkers)
- Supervisor (e.g. clash with supervisors)
- Leadership (e.g. unhappy with senior leadership, unable to adjust to new management style or

organizational direction)

- Discrimination (e.g. not being treated like others)

Burnout.

1. I feel burned out from my work.
2. I worry that this job is hardening me emotionally.

Response options: Never; A few times a year or less; Once a month or less; A few times a month; Once a week; A few times a week; Every day

Discrimination

1. I have experienced discrimination in my workgroup in the past year. (Yes / No)

Culture of well-being^*^

1. I believe my organization has a culture that promotes health and wellness for its staff.
2. I believe the leaders at my organization are actively engaged in role-modeling health and wellness.
3. I think health and wellness programs are readily available to me at my organization.
4. My unit/service line/department regularly supports my participation in health and wellness activities.

Workplace civility^*^

1. People treat each other with respect in my workgroup.
2. Disputes or conflicts are resolved fairly in my workgroup.
3. Discrimination is not tolerated at my workplace.
4. The people I work with cooperate to get the job done.

Workload^*^

1. My workload is reasonable.

Work and family balance^*^

1. My supervisor supports my need to balance work and other life issues

Recognition

1. In my work unit, differences in performance are recognized in a meaningful way.^*^
2. How satisfied are you with the recognition you receive for doing a good job?^±^

Supervisor satisfaction^*^

1. My supervisor listens to what I have to say.
2. My supervisor treats me with respect.
3. I have trust and confidence in my supervisor.
4. My supervisor does not engage in favoritism.
5. It is worthwhile in my workgroup to speak up because something will be done to address our concerns.

Senior leadership^*^

1. In my organization, senior leaders generate high levels of motivation and commitment in the workforce.
2. My organization’s senior leaders maintain high standards of honesty and integrity.
3. Managers communicate the goals of the organization.
4. I have a high level of respect for my organization's senior leaders.
5. Overall, how good a job do you feel is being done by the manager directly above your immediate supervisor? ^±^

Note:

^*^ Response options: Strongly Disagree; Disagree; Neutral; Agree; Strongly Agree

^±^ Response options: Very Dissatisfied; Dissatisfied; Neutral; Satisfied; Very Satisfied
