# Supplementary material for: VA physicians intent to leave and correlations to drivers of burnout: a cross-sectional study
Source: BMC Health Serv Res. 2025 Jan 23;25:125. doi: 10.1186/s12913-024-12079-5 (PMC11755878; doi:10.1186/s12913-024-12079-5)
Supplement: Supplementary file 2 — Supplementary Material 2 [file 12913_2024_12079_MOESM2_ESM.docx]

**Supplemental Appendix B**

**Multinomial regression model for turnover intentions among VA physicians in 2023 (n=11,508).**

|  | **Another VA job** | | **Leave VA** | | | | **Retire** | | | |  |
| --- | --- | --- | --- | --- | --- | --- | --- | --- | --- | --- | --- |
|  | **OR** | **95% CI** | | **OR** | | **95% CI** | | | **OR** | **95% CI** |  |
| Burnout | 2.15* | 1.77 – 2.61 | | 2.97* | | 2.50 – 3.52 | | | 2.45 | 1.97 – 3.05 | |
| Culture of well-being | .94 | .83 – 1.06 | | .83* | | .75 - .93 | | | .99 | .86 – 1.15 | |
| Workplace civility | .98 | .84 – 1.13 | | 1.07 | | .94 – 1.22 | | | .88 | .72 – 1.08 | |
| Discrimination | 2.11* | 1.60 – 2.78 | | 1.90* | | 1.49 – 2.44 | | | .96 | .63 – 1.45 | |
| Reasonable workload | .85* | .78 - .93 | | .85* | | .79 - .91 | | | 1.03 | .93 – 1.14 | |
| Work and family balance | 1.03 | .90 – 1.17 | | 1.03 | | .92 – 1.15 | | | .98 | .83 – 1.16 | |
| Recognition | .81* | .71 - .93 | | .70* | | .63 - .79 | | | .97 | .82 – 1.15 | |
| Supervisor satisfaction | .99 | .98 – 1.00 | | 1.00 | | .99 – 1.00 | | | 1.00 | .99 – 1.01 | |
| Senior leadership | .75* | .66 - .85 | | .64* | | .58 - .72 | | | .80* | .68 - .94 | |
| Gender (ref=female) |  |  | |  | |  | | |  |  | |
| Male | 1.22^*^ | 1.01 – 1.47 | | | 1.58^*^ | 1.35 – 1.87 | | 1.06 | | .86 – 1.32 |  |
| Other | 1.31 | .51 – 3.38 | | | .81 | .31 – 2.14 | | .38 | | .05 – 3.19 |  |
| Age (ref=under 40) |  |  | | |  |  | |  | |  |  |
| 40-49 | 1.00 | .77 – 1.30 | | | .76^*^ | .62 –.93 | | 1.39 | | .36 – 5.34 |  |
| 50-59 | .96 | .74 – 1.25 | | | .44^*^ | .36 –.55 | | 12.26^*^ | | 3.87 – 38.94 |  |
| 60 plus | .60^*^ | .44 – .81 | | | .34^*^ | .26 –.44 | | 136.98^*^ | | 44.09 – 425.59 |  |
| Hispanic (ref non-Hispanic) | .87 | .64 – 1.81 | | | 1.22 | .91 – 1.64 | | 1.05 | | .72 – 1.52 |  |
| Race (ref=White) |  |  | | |  |  | |  | |  |  |
| American Indian or Alaskan Native | .33 | .04 – 2.64 | | | 2.64^*^ | 1.03 – 6.75 | | .29 | | .04 – 2.31 |  |
| Asian | .71^*^ | .56 – .86 | | | .71^*^ | .56 – .91 | | .50^*^ | | .37 –.68 |  |
| Black or African American | 1.19 | .82 – 1.74 | | | .99 | .68 – 1.45 | | .84 | | .52 – 1.35 |  |
| Multi-racial | 1.51 | .86 – 2.63 | | | 1.70^*^ | 1.06 – 2.74 | | .91 | | .37 – 2.24 |  |
| Native Hawaiian or other Pacific Islander | 1.42 | .44 – 4.55 | | | .73 | .19 – 2.79 | | 1.45 | | .38 – 5.53 |  |
| Specialty (ref=primary care) |  |  | | |  |  | |  | |  |  |
| Anesthesiology | .54^*^ | .31 – .91 | | | 1.63^*^ | 1.13 – 2.36 | | 1.16 | | .68 – 1.97 |  |
| Medicine | .70^*^ | .54 – .90 | | | 1.10 | .87 – 1.38 | | 1.10 | | .82 – 1.46 |  |
| Other clinical specialty | .98 | .76 – 1.27 | | | 1.26 | .99 – 1.60 | | 1.29 | | .96 – 1.73 |  |
| Psychiatrist | 1.28 | .97 –1.69 | | | 1.14 | .87 – 1.49 | | 1.24 | | .89 – 1.73 |  |
| Surgery | .67^*^ | .47 – .94 | | | 1.21 | .91 – 1.60 | | .63^*^ | | .44 – .92 |  |
| Faculty (ref=non-faculty) | .91 | .75 – 1.09 | | | 1.18^*^ | 1.00 – 1.39 | | 1.06 | | .86 – 1.30 |  |
| Office | 1.21 | .75 – 1.96 | | | 1.07 | .68 – 1.68 | | .88 | | .48 – 1.62 |  |
| Supervisory role (Ref=None) |  |  | | |  |  | |  | |  |  |
| First line supervisor | 1.26 | .99 – 1.61 | | | 1.18 | .96 – 1.46 | | 1.13 | | .87 – 1.45 |  |
| Manager | 2.30* | 1.82 – 2.90 | | | 1.41* | 1.12 – 1.78 | | 1.10 | | .85 – 1.42 |  |
| MD/population rate | 1.00 | 1.00 - 1.00 | | | 1.00 | 1.00 - 1.00 | | 1.00 | | 1.00 - 1.00 |  |
